# *Caragana korshinskii* Tannin Attenuates LPS-Induced Sheep Rumen Epithelial Injury via TLR4/MyD88/NF-κB Signaling

**DOI:** 10.3390/biology15151258

**Published:** 2026-07-31

**Authors:** Xiaojia Mu, Jiaqi Wang, Deyuan Yao, Huinan Jiang, Shangxiong Zhang, Yuanyuan Xing, Dabiao Li, A La Teng Zhu La

**Affiliations:** 1 Key Laboratory of Animal Nutrition and Feed Science at University of Inner Mongolia Autonomous Region, College of Animal Science, Inner Mongolia Agricultural University, Hohhot 010018, China; 2 National Technology Innovation Center for Prataculture, Hohhot 010018, China

**Keywords:** *Caragana korshinskii* tannin, sheep, rumen epithelial cells, lipopolysaccharide, TLR4/NF-κB signaling, oxidative stress, tight junction

## Abstract

The rumen epithelium mediates nutrient absorption and mucosal defense in ruminants, whereas increased exposure to lipopolysaccharide (LPS) under high-concentrate feeding may induce epithelial inflammation and barrier injury. This study investigated whether *Caragana korshinskii* tannin (CKT) protects against LPS-induced injury in primary sheep rumen epithelial cells and whether TLR4/MyD88/NF-κB signaling is involved. Cells were pretreated with 5 μg/mL CKT for 18 h and then challenged with 5 μg/mL LPS for 6 h. TLR4 knockdown experiments were performed in a sheep rumen epithelial cell line for mechanistic investigation. LPS induced inflammatory cytokine production, oxidative stress, G0/G1 cell-cycle arrest, apoptosis, and reductions in tight-junction-related markers (*p* < 0.05). CKT pretreatment attenuated these changes, as reflected by lower ROS and MDA levels, increased antioxidant enzyme activities, reduced apoptosis, and improved the expression of selected tight-junction-related proteins (*p* < 0.05). CKT also suppressed LPS-recognition components and TLR4/MyD88/NF-κB pathway activation (*p* < 0.05). TLR4 knockdown weakened LPS-induced injury and reduced the additional protective effect of CKT. Overall, CKT may protect rumen epithelial cells against LPS-induced inflammatory injury partly by inhibiting TLR4/MyD88/NF-κB signaling.

## Simple Summary

The rumen epithelium plays an essential role in nutrient absorption and serves as a protective barrier separating rumen contents from underlying body tissues. Disturbance of the rumen environment may expose rumen epithelial cells to bacterial components, such as lipopolysaccharide, thereby inducing epithelial injury. This study investigated whether tannin extracted from *Caragana korshinskii*, a shrub forage resource widely distributed in northern China, could alleviate lipopolysaccharide-induced injury in sheep rumen epithelial cells. In the experiment, sheep rumen epithelial cells were pretreated with the *C. korshinskii* tannin before exposure to lipopolysaccharide to induce inflammatory stress. Tannin pretreatment reduced multiple indicators of cell injury, including inflammatory responses, oxidative damage, and cell apoptosis. Further mechanistic analysis suggested that these protective effects were associated with reduced activation of the Toll-like receptor 4 (TLR4)/myeloid differentiation primary response 88 (MyD88)/nuclear factor kappa B (NF-κB) signaling pathway. Overall, these findings suggest that *C. korshinskii* tannin can attenuate lipopolysaccharide-induced injury in sheep rumen epithelial cells under the tested experimental conditions.

## 1. Introduction

High-concentrate feeding is widely used in intensive sheep production to improve growth performance and production efficiency. The rumen epithelium, as the primary interface between rumen contents and host tissues, mediates nutrient absorption and volatile fatty acid transport and also acts as a physical and immunological barrier [1,2]. However, excessive intake of high-concentrate diets can disturb ruminal fermentation, lower ruminal pH, and promote microbial lysis, leading to increased release of microbe-associated lipopolysaccharide (LPS) [3]. Elevated exposure to LPS and other inflammatory stimuli can compromise rumen epithelial barrier function and promote local inflammatory responses [4,5,6].

As a major component of the outer membrane of Gram-negative bacteria [7], LPS is widely used to establish in vitro models of ruminal epithelial injury. In rumen epithelial cells (RECs), LPS challenge can induce inflammatory cytokine production [8], oxidative stress, and tight junction disruption [9,10]. In goat or bovine rumen epithelial cell models, several plant-derived compounds, including rutin and phloretin, have been reported to attenuate LPS-induced inflammatory and oxidative responses [11,12,13]. These findings support the use of LPS-challenged sheep RECs as a relevant in vitro model for evaluating nutritional bioactives with potential epithelial-protective effects.

The Toll-like receptor 4 (TLR4) is a major pattern-recognition receptor that mediates cellular responses to LPS [14]. Upon LPS exposure, lipopolysaccharide-binding protein (LBP), cluster of differentiation 14 (CD14), and myeloid differentiation factor 2 (MD2)/lymphocyte antigen 96 (LY96) facilitate LPS recognition by TLR4, which subsequently recruits adaptor molecules such as MyD88 [15,16]. Activated NF-κB then drives the expression of pro-inflammatory mediators such as interleukin-1β (IL-1β), interleukin-6 (IL-6), and tumor necrosis factor-α (TNF-α) [17]. Therefore, modulation of TLR4/MyD88/NF-κB signaling may be an important mechanism by which bioactive compounds alleviate LPS-induced inflammatory injury in rumen epithelial cells [18]. Previous studies suggest that dietary bioactives, such as quercetin, can attenuate inflammatory responses in rumen epithelial cells through modulation of the TLR4/NF-κB signaling pathway [19,20].

Plant-derived tannins have gained increasing interest as natural feed bioactives owing to their antioxidant, anti-inflammatory, and microbiota-modulating properties [21]. In ruminants, tannins can influence ruminal fermentation, nitrogen utilization, methane production, and microbial metabolism [22], with effects varying according to their source, chemical structure, and dosage [23]. *Caragana korshinskii* is an important shrub resource in northern China and has been explored as an unconventional forage source for sheep, with reported effects on nutrient utilization and gastrointestinal microbial communities [24]. These findings suggest that *C. korshinskii* and its bioactive components may have potential value in improving rumen function and epithelial health in sheep. Among these bioactive components, *C. korshinskii* tannin (CKT) may be particularly relevant to rumen epithelial protection because tannin-related compounds have been shown to reduce oxidative stress and enhance tight junction integrity in epithelial barrier models [25,26]. Previous studies on tannins in ruminants have mainly focused on their effects on ruminal fermentation, microbial communities, and nutrient utilization, suggesting that their beneficial effects may be mediated, at least in part, through modulation of the rumen microbiota [27]. However, whether CKT itself can directly protect sheep rumen epithelial cells from LPS-induced inflammatory injury remains unclear. In particular, its effects on LPS-induced inflammation, oxidative stress, apoptosis, and tight junction disruption, as well as the involvement of the TLR4/MyD88/NF-κB signaling pathway, have not been fully defined.

We hypothesized that CKT pretreatment could protect sheep RECs against LPS-induced inflammatory, oxidative, apoptotic, and tight-junction-related injury, and that this protective effect would involve modulation of the TLR4/MyD88/NF-κB signaling pathway. Therefore, this study aimed to determine whether CKT attenuates LPS-induced injury in sheep rumen epithelial cells and to assess the involvement of TLR4-mediated NF-κB signaling.

## 2. Materials and Methods

### 2.1. Ethics Statement

All experimental procedures involving animal-derived tissues for the isolation of primary sheep rumen epithelial cells were reviewed and approved by the Animal Care and Use Committee of Inner Mongolia Agricultural University (Approval No. NND2022033; approval date: 7 March 2022). Rumen epithelial tissues were collected from three experimental 3-month-old male Dumeng hybrid sheep. The animals were maintained under standard feeding and management conditions and were fasted for 12 h before slaughter with free access to water. Rumen papillae were isolated from the ventral sac of the rumen immediately after slaughter and transported to the laboratory under sterile and chilled conditions for primary cell isolation. The collection, handling, and use of animal samples were performed in accordance with national and institutional regulations for animal welfare and experimental ethics. All procedures complied with the National Standard Guidelines for Ethical Review of Animal Welfare (GB/T 35892-2018) [28].

### 2.2. Preparation and Purification of Caragana korshinskii Tannin

*C. korshinskii* was harvested from Siziwang Banner, Inner Mongolia Autonomous Region, China, in June 2024. Tannins were extracted from *C. korshinskii* using ultrasonic-assisted solvent extraction. Briefly, the extraction process was optimized using response surface methodology. The optimized conditions were an extraction temperature of 52 °C, an extraction time of 95 min, a liquid-to-solid ratio of 20:1 (mL/g), and 62% aqueous acetone as the extraction solvent. These extraction parameters were determined based on preliminary experiments designed to maximize tannin yield and extraction efficiency, with reference to previously reported methods (Li et al. 2022) [29].

The total condensed tannin content of the crude CKT extract was determined using the phosphotungstic-molybdic acid method and was 265.62 mg/g. To further characterize the chemical composition of the extract, UHPLC-MS/MS-based metabolomic analysis was performed by Wuhan Maiwei Metabolic Biotechnology Co., Ltd. (Wuhan, China). A total of 2556 metabolites were detected, of which 552 were classified as tannin-related compounds. Targeted metabolomic profiling of condensed tannins identified seven monomeric constituents in the crude CKT extract, including epigallocatechin, catechin, gallocatechin gallate, epicatechin, gallocatechin, epicatechin-3-O-gallate, and catechin gallate. Their relative molar proportions were 1:8.88:2.65:1.55:1.92:0.49:0.14, respectively.

To obtain purified CKT for subsequent cell experiments, the crude CKT extract was further purified using HP-20 macroporous adsorption resin. The pretreated crude extract was loaded onto the resin at a concentration of 1.6 mg/mL. Static adsorption was performed at 35 °C for 3 h to allow sufficient binding of tannin components to the resin. The adsorbed tannins were then eluted with 75% ethanol for 3 h. The purification efficiency was evaluated by determining tannin purity before and after resin purification. The tannin purity of the crude extract was 27% before purification and increased to 59% after HP-20 resin purification, with a yield of 16.24%. The purified CKT fraction was used for all subsequent cell culture experiments.

### 2.3. Isolation and Culture of Primary Sheep Rumen Epithelial Cells

Primary sheep rumen epithelial cells were isolated from rumen epithelial tissues collected from the ventral sac of the rumen immediately after slaughter. Briefly, the rumen epithelial tissues were repeatedly rinsed with pre-cooled sterile saline containing antibiotics, including penicillin–streptomycin, amphotericin B, and gentamicin, until the washing solution became clear. The cleaned tissues were then transferred into pre-cooled Dulbecco’s phosphate-buffered saline (DPBS) supplemented with antibiotics and washed thoroughly again. The serosal and muscular layers were bluntly separated using sterile forceps, and the remaining epithelial tissues were cut into small pieces of approximately 4 mm^2^. The tissue fragments were digested with 0.25% trypsin-EDTA, and a single-cell suspension was prepared using a single-cell suspension preparation system (Miltenyi Biotec, Bergisch Gladbach, Germany). The cell suspension was collected and centrifuged at 1000 rpm for 3 min to obtain the cell pellet. The pellet was washed with DPBS and centrifuged under the same conditions; this washing step was repeated three times. The final cell pellet was resuspended in complete culture medium and cultured in a humidified incubator at 37 °C with 5% CO_2_ for one week. Cell attachment and growth were monitored regularly. When cells reached approximately 80% confluence, they were passaged. Differential trypsinization was used to remove fibroblasts and further purify RECs.

RECs were cultured in complete DMEM/F12 medium containing 10% fetal bovine serum, 100 μg/mL streptomycin, 0.25 μg/mL amphotericin B, 50 mg/L gentamicin, 100 mg/L kanamycin, 240 U/mL nystatin, 1% insulin–transferrin–selenium supplement, and 10 ng/mL epidermal growth factor. Cells were maintained at 37 °C in a humidified atmosphere with 5% CO_2_, and the culture medium was refreshed regularly. Cells exhibiting normal morphology and stable growth were selected for subsequent experiments.

### 2.4. Experimental Design for CKT Pretreatment and LPS Challenge

Preliminary dose–response assays were conducted to determine the optimal working concentrations of CKT and LPS based on cell viability and inflammatory cytokine secretion in the culture supernatant, and 5 μg/mL CKT and 5 μg/mL LPS were selected for subsequent experiments.

For the CKT pretreatment and LPS challenge experiment, RECs were assigned to four groups: CON, CKT, LPS, and CKT+LPS. Cells in the CON group were maintained in complete DMEM/F12 medium for 18 h, followed by culture in fresh complete medium for an additional 6 h without additional stimulation. Cells in the CKT group were treated with 5 μg/mL CKT for 18 h, followed by incubation in complete medium for 6 h. Cells in the LPS group were maintained in complete medium for 18 h and then stimulated with 5 μg/mL LPS for 6 h. Cells in the CKT+LPS group were pretreated with 5 μg/mL CKT for 18 h; after pretreatment, the CKT-containing medium was removed, and the cells were washed with sterile PBS before being exposed to fresh complete medium containing 5 μg/mL LPS for 6 h. After treatment, cells and culture supernatants were collected for subsequent analyses.

### 2.5. TLR4 Knockdown in a Sheep Rumen Epithelial Cell Line and Subsequent CKT/LPS Treatment

Primary sheep rumen epithelial cells were used for the main experiments. Because their limited proliferative capacity and difficulty in stable genetic manipulation restricted their use for TLR4 knockdown, a sheep rumen epithelial cell line purchased from Shanghai Liyuan Biotechnology Co., Ltd. (Shanghai, China). was used for mechanistic analysis and cultured under the same basal conditions as described above.

To determine whether TLR4 is involved in the protective effects of CKT against LPS-induced inflammatory injury, TLR4 was knocked down in a sheep rumen epithelial cell line using short hairpin RNA (shRNA) lentiviral particles commercially synthesized by D-Nano Therapeutics (Beijing, China). The TLR4-targeting shRNA sequence was 5′-GAATATGAGTCACAACAAA-3′, and the negative control shRNA sequence was 5′-TTCTCCGAACGTGTCACGT-3′. Before stable TLR4-knockdown cells were established, lentiviral transduction conditions, including the multiplicity of infection and puromycin selection conditions, were optimized in the sheep rumen epithelial cell line (Figure S4). Stable TLR4-knockdown cells were then established, and knockdown efficiency was verified by quantitative real-time PCR and Western blotting (Figure S3A). Following TLR4 knockdown, cells were assigned to five experimental groups: shRNA negative control (Sh-NC), Sh-NC+LPS, TLR4 knockdown (Sh-TLR4), Sh-TLR4+LPS, and Sh-TLR4+CKT+LPS. Cells in the Sh-NC and Sh-TLR4 groups were maintained in complete DMEM/F12 medium for 18 h, followed by culture in fresh complete medium for an additional 6 h without additional stimulation. Cells in the Sh-NC+LPS and Sh-TLR4+LPS groups were cultured in complete medium for 18 h and then exposed to 5 μg/mL LPS for 6 h. Cells in the Sh-TLR4+CKT+LPS group were pretreated with 5 μg/mL CKT for 18 h; after pretreatment, the CKT-containing medium was removed, and the cells were washed with sterile PBS before being exposed to fresh complete medium containing 5 μg/mL LPS for 6 h. After treatment, cells and culture supernatants were collected for determination of inflammatory cytokines, oxidative stress determination, apoptosis-related genes, tight junction markers, and key proteins involved in the TLR4/NF-κB signaling pathway.

### 2.6. Cell Viability Assay

Cell viability was assessed using a Cell Counting Kit-8 assay (CCK-8; SC119, SEVEN Biotechnology, Beijing, China). Briefly, either primary sheep rumen epithelial cells or the sheep rumen epithelial cell line was seeded into culture plates and treated with different concentrations of CKT or LPS. After treatment, CCK-8 reagent was added to each well according to the manufacturer’s instructions, and the plates were incubated at 37 °C for 4 h. Absorbance was then measured at 450 nm using a Multiskan MK3 microplate reader (Thermo Labsystems, Philadelphia, PA, USA). Cell viability was calculated as a percentage of the untreated control group.

### 2.7. Cytokine Measurement by ELISA

After treatment, only the cell culture supernatants were collected, without harvesting the cells. The supernatants were centrifuged at 10,000× *g* for 5 min to remove cell debris, and the clarified supernatants were used for ELISA analysis. The concentrations of IL-1β, IL-6, and TNF-α were determined using commercial ELISA kits according to the manufacturer’s instructions. ELISA kits for IL-1β (BY-ES774009), IL-6 (BY-ES774013), and TNF-α (BY-ES774066) were purchased from BYabscience, Nanjing, China. Absorbance was measured at 450 nm using a Multiskan MK3 microplate reader, and cytokine concentrations were calculated from the corresponding standard curves.

### 2.8. Oxidative Stress Assays

After treatment, intracellular ROS levels and oxidative stress-related indices were assessed. Intracellular ROS levels were detected using a fluorescent probe kit (G0169W, Suzhou Grace Biotechnology Co., Ltd., Suzhou, China) according to the manufacturer’s instructions. Total antioxidant capacity (T-AOC), total superoxide dismutase (T-SOD), catalase (CAT), glutathione peroxidase (GSH-Px), and malondialdehyde (MDA) contents were measured using commercial assay kits. The CAT kit (BC0205) and MDA kit (BC6415) were purchased from Solarbio, Beijing, China, while the T-SOD kit (BC5165), T-AOC kit (A015-2-1), and GSH-Px kit (A005-1) were obtained from Nanjing Jiancheng Bioengineering Institute, Nanjing, China. All assays were performed according to the manufacturers’ protocols.

### 2.9. Flow Cytometry Analysis

Cell-cycle distribution and apoptosis were analyzed by flow cytometry. After the corresponding treatments, RECs were harvested and washed twice with cold PBS. For cell-cycle analysis, cells were fixed with pre-cooled 70% ethanol at 4 °C for 24 h. After fixation, the cells were washed with PBS to remove residual ethanol and stained using a cell cycle and apoptosis analysis kit (Beyotime, Shanghai, China, C1052) according to the manufacturer’s instructions. The DNA content was detected by flow cytometry, and the proportions of cells in the G0/G1, S, and G2/M phases were calculated. For apoptosis analysis, cells were stained using an Annexin V-FITC apoptosis detection kit (C1062, Beyotime Biotechnology, Shanghai, China). Briefly, harvested cells were washed twice with cold PBS and resuspended in binding buffer. The cells were then incubated with Annexin V-FITC and propidium iodide in the dark according to the manufacturer’s protocol. Apoptotic cells were detected by flow cytometry, and the proportions of early apoptotic, late apoptotic, and total apoptotic cell populations were quantified.

### 2.10. Quantitative Real-Time PCR

Total RNA was extracted from treated RECs using a Total RNA Extraction Kit (SM132, SEVEN Biotechnology, Beijing, China,) according to the manufacturer’s protocol. RNA concentration and purity were determined using a spectrophotometer (Thermo Fisher Scientific, Waltham, MA, USA). Complementary DNA was synthesized using a Reverse Transcription Kit (AG, Changsha, China, 11728). Quantitative real-time PCR was performed to determine the mRNA expression levels of inflammatory cytokines, antioxidant-related genes, apoptosis-related genes, tight-junction-related genes, and TLR4 signaling pathway-related genes. Relative gene expression was calculated using the 2^−ΔΔCt^ method, with ACTB (β-actin) used as the internal reference gene. The primer sequences used for quantitative real-time PCR are listed in Table S1.

### 2.11. Western Blot Analysis

Total protein was extracted from treated cells using RIPA lysis buffer (SW205, SEVEN Biotechnology) supplemented with a protease and phosphatase inhibitor cocktail. To assess NF-κB p65 nuclear translocation, nuclear and cytoplasmic proteins were isolated separately. Protein concentrations were determined using a BCA protein assay kit (SW101-02, SEVEN Biotechnology). Equal amounts of protein were separated by SDS-PAGE and transferred onto PVDF membranes. After blocking, the membranes were incubated with primary antibodies against TLR4, MyD88, IKK, p-IKK, IκBα, p-IκBα, NF-κB p65, p-NF-κB p65, ZO-1, OCLN, CLDN1, ACTB, and Lamin B1, followed by incubation with fluorescent dye-conjugated secondary antibodies. Information on the antibodies used for Western blot analysis is provided in Table S2. Protein bands were detected using a fluorescence imaging system and quantified with image analysis software. ACTB was used as the loading control for total and cytoplasmic proteins, and Lamin B1 was used as the loading control for nuclear proteins. Target protein abundance was normalized to the corresponding internal control. All available original Western blot membrane-section images, including the complete available membrane regions, sample lanes, and retained molecular-weight marker lanes, are provided in Figures S5–S10.

### 2.12. Immunofluorescence Staining

Immunofluorescence staining was performed to identify RECs and assess the cellular localization of TLR4 and NF-κB p65. Briefly, cells grown on sterile coverslips were washed three times with PBS and fixed with 4% paraformaldehyde for 15 min at room temperature. After washing with PBS, cells were permeabilized with 0.1% Triton X-100 in PBS for 10 min and then blocked with 5% bovine serum albumin (BSA) in PBS for 1 h at room temperature. For REC identification, cells were incubated overnight at 4 °C with a primary antibody against CK18 (10830-1-AP, Proteintech, Wuhan, China; 1:200). After three washes with PBS, cells were incubated with a fluorophore-conjugated goat anti-rabbit IgG secondary antibody (1:500) for 1 h at room temperature in the dark. Nuclei were counterstained with DAPI for 5 min. For TLR4 and NF-κB p65 localization analysis, cells were incubated overnight at 4 °C with primary antibodies against TLR4 (19811-1-AP, Proteintech, Wuhan, China; 1:200) and NF-κB p65 (10745-1-AP, Proteintech, Wuhan, China; 1:200). After washing with PBS, cells were incubated with the corresponding fluorophore-conjugated goat anti-rabbit IgG secondary antibodies (1:500) for 1 h at room temperature in the dark. Nuclei were stained with DAPI for 5 min. Fluorescence images were captured using a fluorescence microscope (Leica DMi8, Leica Microsystems, Wetzlar, Germany) under identical exposure settings within each experiment.

### 2.13. Statistical Analysis

All statistical analyses were performed using SPSS 19.0 software (SPSS Inc., Chicago, IL, USA). Data are presented as means ± SEM. The number of biological or technical replicates for each assay is indicated in the corresponding figure legends. Unless otherwise specified, cell viability, ELISA, oxidative stress indices, ROS fluorescence intensity, flow cytometry, immunofluorescence quantification, and qRT-PCR analyses were performed with six replicates, whereas Western blot analyses were performed with three replicates. Before statistical comparisons, data were examined for normal distribution and homogeneity of variance. For normally distributed data with homogeneous variance, differences among multiple groups were analyzed using one-way analysis of variance (ANOVA), followed by Tukey’s post hoc multiple-comparison test. When the assumption of homogeneity of variance was not met, Welch’s ANOVA followed by Games–Howell post hoc test was applied. A value of *p* < 0.05 was considered statistically significant. Different lowercase letters above the bars indicate significant differences among groups.

## 3. Results

### 3.1. Identification of Primary Rumen Epithelial Cells

Primary rumen epithelial cells were identified by immunofluorescence staining for CK18, a marker of epithelial cells. CK18-positive fluorescence was observed in the cytoplasm, and DAPI staining showed intact nuclei. The merged images showed epithelial-like morphology, and CK18 staining together with cell morphology supported the epithelial identity of the isolated primary sheep rumen epithelial cells (Figure S1).

### 3.2. Determination of Optimal Working Concentrations of LPS and CKT

**Figure 1:**
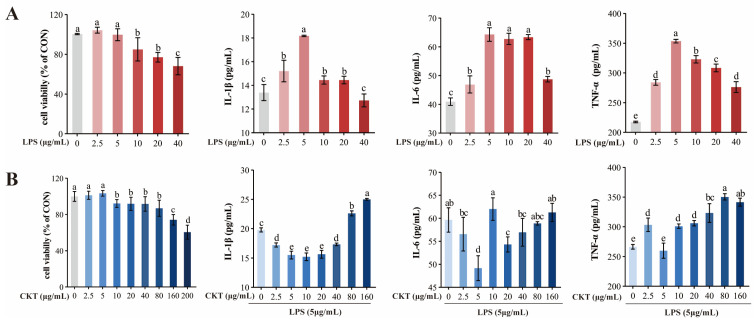
Selection of working concentrations of LPS and CKT in primary sheep rumen epithelial cells. (**A**) Effects of different concentrations of lipopolysaccharide (LPS: 0, 2.5, 5, 10, 20, and 40 μg/mL) on cell viability and the secretion of inflammatory cytokines in primary sheep rumen epithelial cells. Red bars represent the LPS concentration series. (**B**) Effects of different concentrations of *Caragana korshinskii* tannin (CKT: 0, 2.5, 5, 10, 20, 40, 80, 160, and/or 200 μg/mL) on cell viability and inflammatory cytokine secretion in LPS-challenged primary sheep rumen epithelial cells. Blue bars represent the CKT concentration series. Data are presented as means ± SEM (*n* = 6). Bars with different lowercase letters indicate significant differences among groups (*p* < 0.05).

Cell viability and inflammatory cytokine secretion were evaluated to determine the optimal working concentrations of LPS and CKT. LPS at 2.5 and 5 μg/mL did not significantly affect REC viability, whereas 10, 20, and 40 μg/mL LPS significantly reduced cell viability (*p* < 0.05; Figure 1A). Meanwhile, LPS increased IL-1β, IL-6, and TNF-α secretion, with 5 μg/mL LPS inducing a strong inflammatory response without detectable cytotoxicity. Thus, 5 μg/mL LPS was selected for subsequent experiments. For CKT, 2.5 and 5 μg/mL showed no obvious cytotoxicity, whereas concentrations ≥ 10 μg/mL significantly reduced cell viability, with more pronounced effects at 160 and 200 μg/mL (*p* < 0.05; Figure 1B). Under LPS stimulation, 5 μg/mL CKT most consistently reduced IL-1β, IL-6, and TNF-α levels without affecting cell viability (*p* < 0.05; Figure 1B). Therefore, 5 μg/mL CKT was selected as the pretreatment concentration for subsequent experiments.

### 3.3. CKT Alleviated LPS-Induced Cell-Cycle Arrest and Apoptosis in Rumen Epithelial Cells

**Figure 2:**
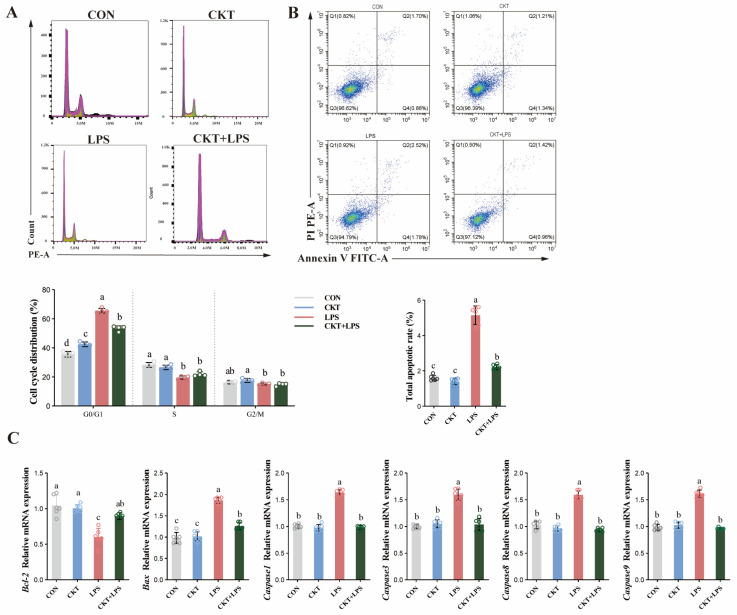
CKT alleviates LPS-induced cell-cycle arrest and apoptosis in primary sheep rumen epithelial cells. (**A**) Representative flow cytometry histograms of cell-cycle distribution and quantitative analysis of the proportions of cells in the G0/G1, S, and G2/M phases. (**B**) Representative Annexin V-FITC/PI flow cytometry plots and quantitative analysis of the total apoptotic rate. The total apoptotic rate was calculated as the sum of early and late apoptotic cells. The colors in panels A and B are generated by the flow-cytometry analysis software to visualize the fitted cell-cycle distributions and event density, respectively, and do not represent different treatment groups. In the flow-cytometry histograms, K and M denote thousands and millions, respectively. (**C**) Relative mRNA expression of apoptosis-related genes, including *Bcl-2*, *Bax*, *Caspase1*, *Caspase3*, *Caspase8*, and *Caspase9*. Data are presented as means ± SEM (*n* = 6). Gray, blue, red, and green bars represent the CON, CKT, LPS, and CKT+LPS groups, respectively. Bars with different lowercase letters indicate significant differences among groups (*p* < 0.05).

Flow cytometric analysis showed that LPS stimulation significantly altered cell-cycle distribution in rumen epithelial cells. Compared with the CON group, the LPS group had a significantly higher proportion of cells in the G0/G1 phase and a lower proportion in the S phase, indicating G0/G1-phase arrest (*p* < 0.05; Figure 2A). CKT pretreatment partly reversed these changes, as evidenced by a significant decrease in the G0/G1-phase cell population in the CKT+LPS group compared with the LPS group (*p* < 0.05; Figure 2A). Annexin V-FITC/PI staining further showed that LPS significantly increased the proportion of apoptotic cells, whereas CKT pretreatment significantly reduced apoptosis (*p* < 0.05; Figure 2B). LPS also significantly decreased the expression of the anti-apoptotic gene *Bcl-2* and increased the expression of the pro-apoptotic genes *Bax* and *Caspase1/3/8/9* (*p* < 0.05; Figure 2C). In contrast, CKT pretreatment significantly restored *Bcl-2* expression and suppressed the LPS-induced upregulation of these pro-apoptotic genes (*p* < 0.05; Figure 2C). Together, these results indicate that CKT alleviated LPS-induced cell-cycle arrest and apoptotic injury in rumen epithelial cells.

### 3.4. CKT Reduced LPS-Induced Oxidative Stress in Rumen Epithelial Cells

**Figure 3:**
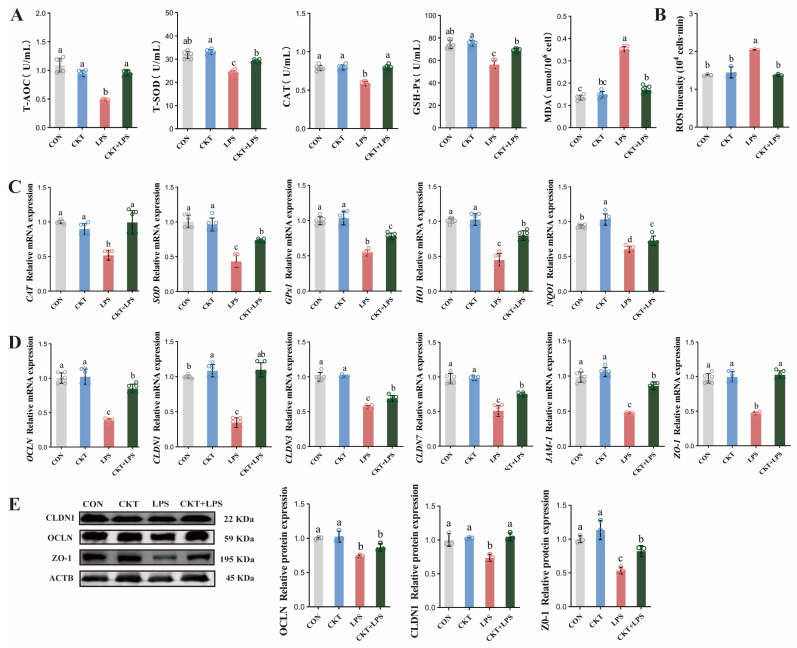
Effects of CKT on oxidative stress and tight-junction-related molecular markers in LPS-challenged primary sheep rumen epithelial cells. (**A**) Antioxidant capacity and lipid peroxidation-related indices, including T-AOC, T-SOD, CAT, GSH-Px, and MDA. (**B**) Quantitative analysis of intracellular ROS intensity. (**C**) Relative mRNA expression of antioxidant-related genes, including *CAT*, *SOD*, *GPx1*, *HO1*, and *NQO1*. (**D**) Relative mRNA expression of tight-junction-related genes, including *OCLN*, *CLDN1*, *CLDN3*, *CLDN7*, *JAM-1*, and *ZO-1*. (**E**) Representative Western blot bands and quantitative analysis of OCLN, CLDN1, and ZO-1 protein abundance. ACTB was used as the loading control. Data are presented as means ± SEM. For biochemical assays, ROS analysis, and qRT-PCR, *n* = 6; for Western blot analysis, *n* = 3. Gray, blue, red, and green bars represent the CON, CKT, LPS, and CKT+LPS groups, respectively. Bars with different lowercase letters indicate significant differences among groups (*p* < 0.05).

LPS stimulation significantly induced oxidative stress in rumen epithelial cells. Compared with the CON group, the LPS group showed significantly decreased T-AOC levels and reduced activities of T-SOD, CAT, and GSH-Px, accompanied by a significant increase in MDA content (*p* < 0.05; Figure 3A). ROS fluorescence intensity was also significantly increased after LPS stimulation (*p* < 0.05; Figure 3B). In parallel, the mRNA expression levels of antioxidant-related genes, including *CAT*, *SOD*, *GPx1*, *HO1*, and *NQO1*, were significantly downregulated in the LPS group compared with the CON group (*p* < 0.05; Figure 3C). Compared with the LPS group, CKT pretreatment significantly increased T-AOC level and the activities of T-SOD, CAT, and GSH-Px, while significantly decreasing MDA content and ROS intensity in the CKT+LPS group (*p* < 0.05; Figure 3A,B). Consistently, CKT pretreatment significantly upregulated the mRNA expression of *CAT*, *SOD*, *GPx1*, *HO1*, and *NQO1* compared with the LPS group (*p* < 0.05; Figure 3C). These results indicate that CKT partially restored antioxidant capacity and alleviated LPS-induced oxidative stress, as reflected by improved antioxidant-related indicators and reduced lipid peroxidation and ROS accumulation.

### 3.5. CKT Partially Restored Tight-Junction-Related Molecular Markers in LPS-Challenged Rumen Epithelial Cells

LPS stimulation significantly impaired epithelial barrier-related markers in rumen epithelial cells. Compared with the CON group, the LPS group showed significantly decreased mRNA expression of *OCLN*, *CLDN1/3/7*, *JAM1*, and *ZO-1*, together with reduced protein abundance of OCLN, CLDN1, and ZO-1 (*p* < 0.05; Figure 3D,E). In contrast, CKT pretreatment significantly restored the mRNA expression of tight-junction-related genes and increased the protein abundance of CLDN1 and ZO-1 in LPS-stimulated cells (*p* < 0.05; Figure 3D,E). FITC-phalloidin staining showed no obvious difference in actin organization among groups (Figure S2). Together, these findings indicate that CKT partially alleviated the LPS-induced reduction in tight-junction-related molecular markers, as reflected by the restoration of tight-junction-related gene expression and partial improvement in tight junction protein abundance.

### 3.6. CKT Inhibited LPS-Induced Activation of the TLR4/MyD88/NF-κB Signaling Pathway

**Figure 4:**
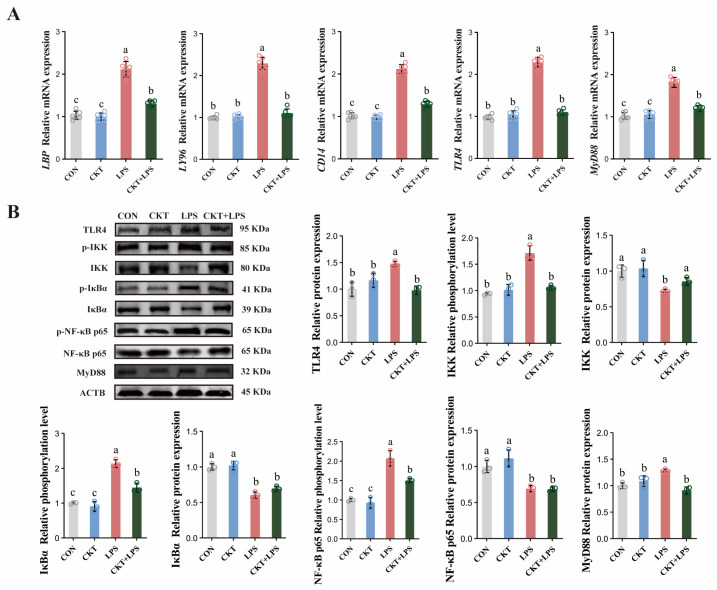
CKT suppresses LPS-induced activation of the TLR4/MyD88/NF-κB signaling pathway in primary sheep rumen epithelial cells. (**A**) Relative mRNA expression levels of *LBP*, *LY96*, *CD14*, *TLR4*, and *MyD88*. (**B**) Representative Western blot bands and quantitative analysis of TLR4, p-IKK, IKK, p-IκBα, IκBα, p-NF-κB p65, NF-κB p65, and MyD88 protein expression. Data are presented as means ± SEM. For qRT-PCR analysis, *n* = 6; for Western blot analysis, *n* = 3. Gray, blue, red, and green bars represent the CON, CKT, LPS, and CKT+LPS groups, respectively. Bars with different lowercase letters indicate significant differences among groups (*p* < 0.05).

**Figure 5:**
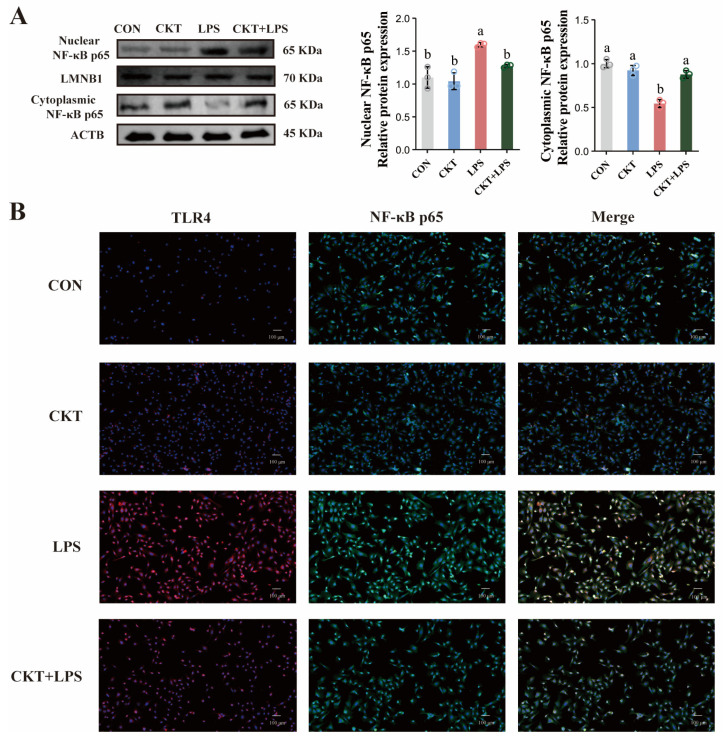
CKT inhibits NF-κB p65 nuclear translocation induced by LPS in primary sheep rumen epithelial cells. (**A**) Representative Western blot bands and quantitative analysis of nuclear and cytoplasmic NF-κB p65. Lamin B1 (LMNB1) and ACTB were used as loading controls for nuclear and cytoplasmic proteins, respectively. (**B**) Representative immunofluorescence images showing the localization of TLR4 and NF-κB p65. Nuclei were counterstained with DAPI. Scale bars = 100 μm. Data are presented as means ± SEM. For Western blot analysis, *n* = 3. Gray, blue, red, and green bars represent the CON, CKT, LPS, and CKT+LPS groups, respectively. Bars with different lowercase letters indicate significant differences among groups (*p* < 0.05).

To elucidate the mechanism underlying the anti-inflammatory effect of CKT, the TLR4/MyD88/NF-κB signaling pathway was examined at both the transcriptional and protein levels. Compared with the CON group, LPS stimulation significantly upregulated the mRNA expression of *LBP*, *LY96*, *CD14*, *TLR4*, and *MyD88* and increased the protein abundance of TLR4 and MyD88 (*p* < 0.05; Figure 4A,B). Meanwhile, LPS significantly enhanced the phosphorylation of IKK, IκBα, and NF-κB p65, accompanied by reduced IκBα protein abundance, indicating activation of the TLR4/MyD88/NF-κB signaling cascade (*p* < 0.05; Figure 4B). Consistently, subcellular protein analysis showed that LPS significantly increased nuclear NF-κB p65 accumulation and decreased cytoplasmic NF-κB p65 levels, indicating increased NF-κB p65 nuclear translocation (*p* < 0.05; Figure 5A). Compared with the LPS group, CKT pretreatment significantly downregulated the mRNA expression of pathway-related genes, reduced TLR4 and MyD88 protein abundance, inhibited IKK, IκBα, and NF-κB p65 phosphorylation, and preserved IκBα protein abundance (*p* < 0.05; Figure 4A,B). CKT pretreatment also significantly reduced nuclear NF-κB p65 accumulation and partially restored cytoplasmic NF-κB p65 abundance compared with the LPS group (*p* < 0.05; Figure 5A). Immunofluorescence staining further supported these findings. Compared with the CON group, LPS stimulation was associated with increased TLR4 fluorescence intensity and more evident NF-κB p65 nuclear localization, whereas CKT pretreatment reduced TLR4 fluorescence intensity and attenuated NF-κB p65 nuclear localization (Figure 5B). Collectively, these results indicate that CKT suppressed LPS-induced inflammatory signaling in rumen epithelial cells by inhibiting activation of the TLR4/MyD88/NF-κB pathway.

### 3.7. TLR4 Knockdown Indicates That the Protective Effect of CKT Is Associated with TLR4-Mediated Signaling

To further examine whether TLR4-mediated signaling was involved in the protective effect of CKT, TLR4 knockdown was performed in a sheep rumen epithelial cell line. This experiment was designed as a mechanistic verification of the TLR4/MyD88/NF-κB pathway rather than a direct repetition of the primary-cell experiments. Both qRT-PCR and Western blot analyses confirmed that TLR4 mRNA and protein expression were markedly reduced in the Sh-TLR4 group compared with the Sh-NC group (*p* < 0.05; Figure S3A), indicating successful TLR4 knockdown. Cell viability was not significantly affected by TLR4 knockdown or the subsequent treatments (*p* > 0.05; Figure S3B), indicating that the observed cellular responses were unlikely to result from differences in cell viability.

**Figure 6:**
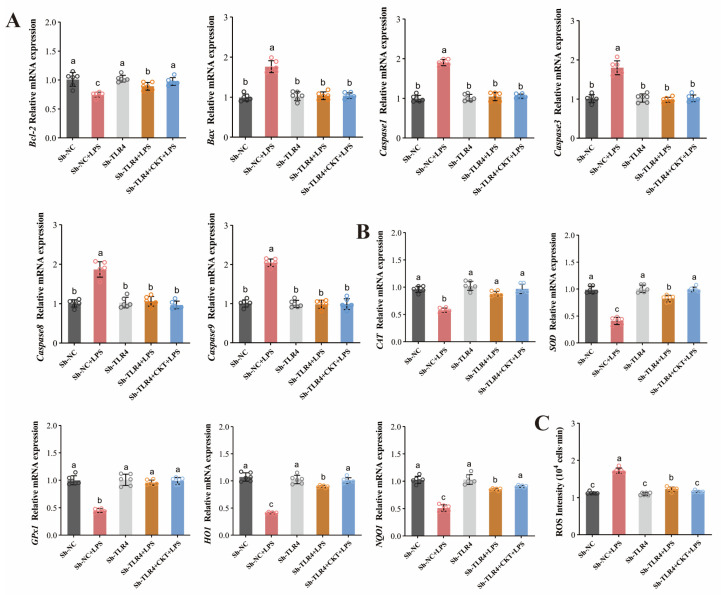
TLR4 knockdown attenuates LPS-induced apoptosis-related and oxidative stress responses in a sheep rumen epithelial cell line. (**A**) Relative mRNA expression of apoptosis-related genes, including *Bcl-2*, *Bax*, *Caspase1*, *Caspase3*, *Caspase8*, and *Caspase9*. (**B**) Relative mRNA expression of antioxidant-related genes, including *CAT*, *SOD*, *GPx1*, *HO1*, and *NQO1*. (**C**) Quantitative analysis of intracellular ROS intensity. Data are presented as means ± SEM (*n* = 6). Dark gray, red, light gray, orange, and blue bars represent the Sh-NC, Sh-NC+LPS, Sh-TLR4, Sh-TLR4+LPS, and Sh-TLR4+CKT+LPS groups, respectively. Bars with different lowercase letters indicate significant differences among groups (*p* < 0.05). TLR4 knockdown also weakened LPS-induced inflammatory activation and LPS-recognition signaling in a sheep rumen epithelial cell line. Compared with the Sh-NC+LPS group, the Sh-TLR4+LPS group exhibited significantly lower concentrations of IL-1β, IL-6, and TNF-α, together with reduced mRNA expression of inflammatory cytokines and LPS-recognition-related genes, including *IL-1β*, *IL-6*, *TNF-α*, *LBP*, *CD14*, *LY96*, *TLR4*, and *MyD88* (*p* < 0.05; Figure 7A,B). Importantly, under the TLR4-knockdown background, CKT pretreatment did not further significantly suppress most inflammatory cytokines or LPS-recognition-related genes compared with the Sh-TLR4+LPS group (*p* > 0.05; Figure 7A,B). This result indicates that once TLR4-mediated recognition and downstream signal transduction were weakened, the additional anti-inflammatory effect of CKT was largely diminished. Therefore, the inhibitory effect of CKT on LPS-induced inflammatory responses may be closely associated with suppression of the TLR4/MyD88-mediated inflammatory signaling pathway.

Under LPS stimulation, TLR4 knockdown reduced LPS-induced apoptotic and oxidative responses in the sheep rumen epithelial cell line. Compared with the Sh-NC+LPS group, the Sh-TLR4+LPS group showed increased *Bcl-2* expression and decreased expression of *Bax* and *Caspase1/3/8/9* (*p* < 0.05; Figure 6A). In addition, TLR4 knockdown partially restored the expression of antioxidant-related genes, including *CAT*, *SOD*, *GPx1*, *HO1*, and *NQO1*, and reduced intracellular ROS accumulation (*p* < 0.05; Figure 6B,C). Notably, when CKT was added under the TLR4-knockdown background, the expression of most apoptosis-related and antioxidant-related genes showed no further marked change compared with the Sh-TLR4+LPS group, although ROS accumulation was further reduced. These results suggest that TLR4 knockdown weakened LPS-induced oxidative and apoptotic injury, and that the protective effect of CKT was partly diminished after inhibition of TLR4 signaling.

**Figure 7:**
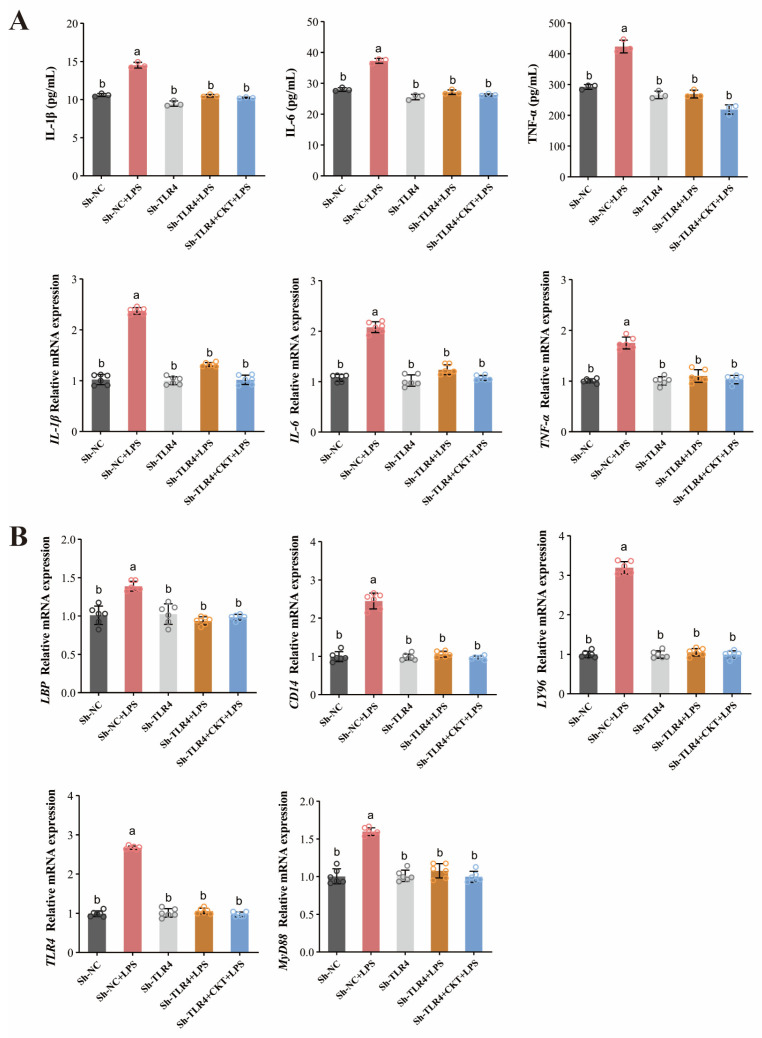
TLR4 knockdown attenuates LPS-induced inflammatory responses and LPS-recognition signaling in a sheep rumen epithelial cell line. (**A**) Concentrations of IL-1β, IL-6, and TNF-α in the culture supernatant, and relative mRNA expression of *IL-1β*, *IL-6*, and *TNF-α*. (**B**) Relative mRNA expression of LPS-recognition and TLR4 pathway-related genes, including *LBP*, *CD14*, *LY96*, *TLR4*, and *MyD88*. Data are presented as means ± SEM. For ELISA analysis, *n* = 6; for qRT-PCR analysis, *n* = 6. Dark gray, red, light gray, orange, and blue bars represent the Sh-NC, Sh-NC+LPS, Sh-TLR4, Sh-TLR4+LPS, and Sh-TLR4+CKT+LPS groups, respectively. Bars with different lowercase letters indicate significant differences among groups (*p* < 0.05).

### 3.8. TLR4 Knockdown Supports the Involvement of TLR4/MyD88/NF-κB Signaling in the Protective Effect of CKT

**Figure 8:**
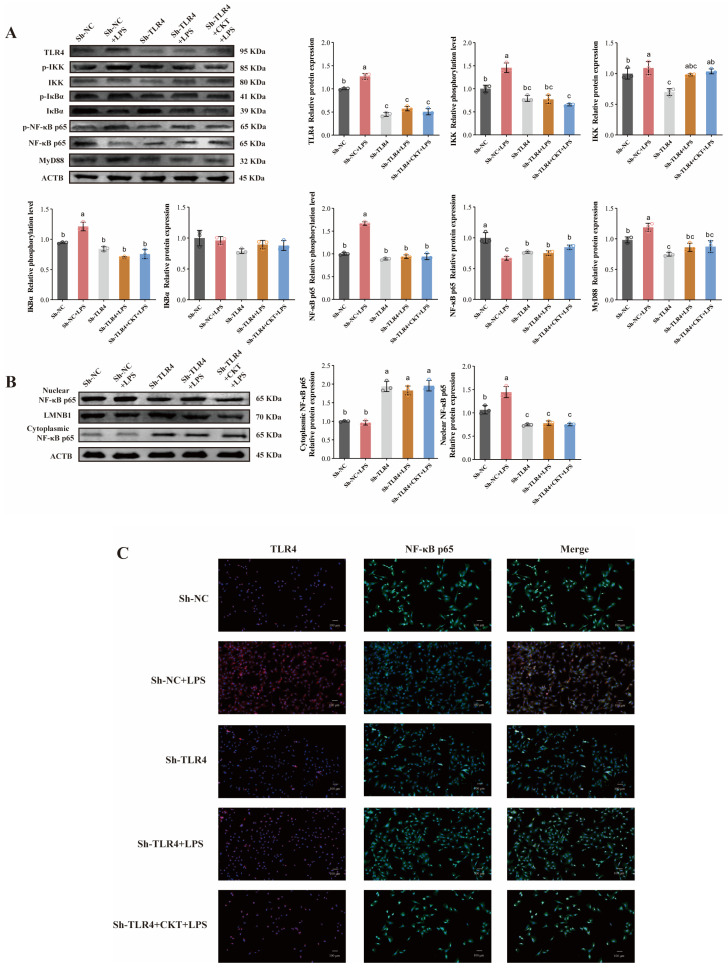
TLR4 knockdown supports the involvement of the TLR4/NF-κB axis in the protective effect of CKT in a sheep rumen epithelial cell line. (**A**) Representative Western blot bands and quantitative analysis of TLR4/NF-κB pathway-related proteins. (**B**) Representative Western blot bands and quantitative analysis of nuclear and cytoplasmic NF-κB p65. LMNB1 and ACTB were used as loading controls for nuclear and cytoplasmic proteins, respectively. (**C**) Representative immunofluorescence images showing the localization of TLR4 and NF-κB p65. Nuclei were counterstained with DAPI. Scale bars = 100 μm. Data are presented as means ± SEM (*n* = 3). Dark gray, red, light gray, orange, and blue bars represent the Sh-NC, Sh-NC+LPS, Sh-TLR4, Sh-TLR4+LPS, and Sh-TLR4+CKT+LPS groups, respectively. Bars with different lowercase letters indicate significant differences among groups (*p* < 0.05).

To further determine whether the protective effect of CKT was associated with TLR4-mediated NF-κB signaling, pathway activation was examined in the TLR4 knockdown sheep rumen epithelial cell line. Western blot analysis showed that LPS significantly activated the TLR4/MyD88/NF-κB signaling pathway in Sh-NC cells, as reflected by increased TLR4 and MyD88 protein abundance and enhanced phosphorylation of IKK, IκBα, and NF-κB p65 (*p* < 0.05; Figure 8A). After TLR4 knockdown, however, LPS-induced activation of this pathway was significantly weakened, with reduced TLR4 and MyD88 expression and lower phosphorylation levels of IKK, IκBα, and NF-κB p65 (*p* < 0.05; Figure 8A). Notably, under the TLR4-knockdown background, CKT pretreatment did not further consistently suppress most TLR4/MyD88/NF-κB pathway-related proteins compared with the Sh-TLR4+LPS group (*p* > 0.05; Figure 8A).

Subcellular fractionation further showed that TLR4 knockdown inhibited LPS-induced NF-κB p65 nuclear translocation in the sheep rumen epithelial cell line, as indicated by decreased nuclear NF-κB p65 levels and increased cytoplasmic NF-κB p65 levels (*p* < 0.05; Figure 8B). Consistently, immunofluorescence staining showed strong TLR4 expression and prominent NF-κB p65 nuclear localization in Sh-NC+LPS cells, whereas both signals were reduced after TLR4 knockdown. In the Sh-TLR4+CKT+LPS group, TLR4 and NF-κB p65 staining showed a similar inhibitory pattern to that observed in the Sh-TLR4+LPS group (Figure 8C). Together, these results indicate that TLR4 knockdown weakened LPS-induced TLR4/MyD88/NF-κB signaling activation and largely diminished the additional inhibitory effect of CKT, suggesting that the protective effect of CKT against LPS-induced epithelial injury is closely associated with suppression of the TLR4-mediated NF-κB signaling pathway.

## 4. Discussion

The rumen epithelium is continuously exposed to fermentation products, microbial metabolites, and microbe-associated molecular patterns and therefore represents an important interface for maintaining ruminal homeostasis [30,31].

Under high-concentrate feeding conditions, increased ruminal LPS exposure may trigger epithelial inflammation and cellular injury [32]. In the present study, CKT pretreatment alleviated LPS-induced damage in sheep rumen epithelial cells, suggesting that this tannin-rich fraction may exert a protective effect against inflammatory stress at the epithelial level. However, the purified CKT used in this study should be regarded as a tannin-rich phytochemical fraction rather than a single purified compound. Although HP-20 resin purification increased tannin purity from 27% to 59%, non-tannin constituents may still have remained in the purified fraction. Seven tannin-related monomeric constituents were identified, including epigallocatechin, catechin, gallocatechin gallate, epicatechin, gallocatechin, epicatechin-3-O-gallate, and catechin gallate. Therefore, the observed protective effect is more appropriately attributed to the purified CKT fraction as a whole, and possible additive or synergistic interactions among its tannin-related and other co-purified constituents cannot be excluded.

The TLR4/MyD88/NF-κB pathway appears to be a central signaling mechanism underlying the protective effect of CKT. LPS recognition by the TLR4 receptor complex promotes the recruitment of MyD88 and subsequent activation of the IKK/IκBα signaling cascade. Phosphorylation of IκBα facilitates NF-κB p65 nuclear translocation and increases the transcription of pro-inflammatory cytokines, including IL-1β, IL-6, and TNF-α [17]. In the present study, LPS enhanced the expression of LPS-recognition-related molecules, activated TLR4/MyD88 signaling, promoted IKK, IκBα, and NF-κB p65 phosphorylation, and increased NF-κB p65 nuclear accumulation. CKT pretreatment attenuated these changes and reduced inflammatory cytokine production. These findings indicate that CKT may suppress both the upstream recognition of LPS and the downstream amplification of NF-κB-dependent inflammatory signaling. This interpretation is consistent with previous evidence that plant-derived tannins and polyphenols can alleviate LPS-induced inflammatory responses by modulating NF-κB-related pathways [33,34,35,36,37].

Suppression of inflammatory signaling was accompanied by coordinated improvements in oxidative stress, cell-cycle progression, apoptosis, and tight-junction-related molecular markers. Inflammatory activation can promote excessive ROS generation, while accumulated ROS may further enhance redox-sensitive inflammatory signaling, including NF-κB activation, thereby forming a self-amplifying interaction between inflammation and oxidative stress [34,38]. Excessive ROS and lipid peroxidation may subsequently disturb cell-cycle progression, promote apoptotic cell loss, and impair epithelial renewal and tight junction regulation. In the present study, CKT reduced ROS and MDA accumulation, partially restored antioxidant enzyme activities and antioxidant-related gene expression, alleviated G0/G1-phase arrest, and reduced apoptosis. CKT also partially restored the expression of OCLN, CLDN1, and ZO-1, indicating partial preservation of tight-junction-related molecular markers under LPS-induced stress. The antioxidant-related effects of CKT may partly be associated with the phenolic hydroxyl groups and galloyl moieties present in its tannin-related constituents, which have been linked to electron or hydrogen donation, metal-ion chelation, and reduced ROS accumulation and lipid peroxidation [39,40,41,42]. However, because direct radical-scavenging activity and upstream antioxidant signaling were not assessed, the present study cannot distinguish whether CKT acted through direct antioxidant activity, enhancement of endogenous antioxidant defenses, or both.

The TLR4 knockdown experiment provided further mechanistic support for this interpretation. Under LPS challenge, TLR4 knockdown reduced inflammatory cytokine production, oxidative stress, apoptosis-related responses, and activation of the NF-κB pathway. Moreover, once TLR4 expression was suppressed, the additional protective effect of CKT was markedly reduced for most measured outcomes. This pattern suggests that TLR4-mediated signaling is an important upstream target associated with CKT-mediated protection. The use of a stable sheep rumen epithelial cell line for the knockdown experiment was necessary because primary rumen epithelial cells have limited proliferative capacity and are difficult to expand through repeated lentiviral transduction, antibiotic selection, and stable gene knockdown. Similar strategies have been used in previous rumen epithelial studies, in which stable or immortalized rumen epithelial cell lines and receptor-knockdown models were employed to investigate receptor-dependent regulation of LPS-induced inflammatory responses [8,43]. Nevertheless, the residual effects of CKT after TLR4 knockdown also suggest that CKT is unlikely to act exclusively through this pathway, because tannin-related polyphenols may simultaneously regulate other inflammatory and redox-sensitive signaling networks [42,44].

These findings should be interpreted within the scope of the present cell-based study. Primary sheep rumen epithelial cells were used to evaluate the principal protective effects of CKT, whereas a stable cell line was used specifically for mechanistic knockdown analysis. Therefore, the knockdown results support the involvement of TLR4/MyD88/NF-κB signaling but require further confirmation in primary rumen epithelial cells and in vivo sheep models. CK18 staining and epithelial-like morphology supported the epithelial identity of the isolated primary cells, although additional epithelial and mesenchymal markers would provide more comprehensive characterization. Likewise, changes in OCLN, CLDN1, and ZO-1 represent tight-junction-related molecular evidence rather than direct demonstration of barrier function. Future studies should therefore combine primary-cell validation, functional barrier assays, rumen tissue immunohistochemistry or immunofluorescence, and sheep feeding trials. Such studies will be necessary to determine whether the cellular effects observed here can be reproduced in vivo and to establish the effective dietary dose, safety, ruminal availability, and practical value of CKT supplementation.

## 5. Conclusions

In conclusion, CKT alleviated LPS-induced injury in sheep rumen epithelial cells, and this effect was closely associated with suppression of the TLR4/MyD88/NF-κB signaling pathway. This pathway inhibition was accompanied by reduced inflammatory cytokine production and oxidative stress, thereby helping to relieve cell-cycle arrest, apoptosis-related injury, and the decline in tight-junction-related molecules. TLR4 knockdown experiments in a sheep rumen epithelial cell line further supported the involvement of TLR4-mediated NF-κB signaling in CKT-mediated protection. These findings suggest that CKT has the potential to support rumen epithelial homeostasis under LPS-induced inflammatory stress. Further validation under in vivo feeding conditions is needed to establish the physiological relevance and practical implications of these cellular findings.

## Data Availability

The data presented in this study are available within the article and Supplementary Materials. Additional data supporting the findings of this study are available from the corresponding authors upon reasonable request.

## Supplementary Materials

The following supporting information can be downloaded at https://www.mdpi.com/article/10.3390/biology15151258/s1. Figure S1: CK18 immunofluorescence staining supporting the epithelial identity of isolated primary sheep rumen epithelial cells; Figure S2: Effects of CKT pretreatment on F-actin organization in LPS-challenged primary sheep rumen epithelial cells; Figure S3: Verification of TLR4 knockdown efficiency and effects on cell viability in a sheep rumen epithelial cell line; Figure S4: Optimization of lentiviral transduction conditions and puromycin selection for stable TLR4-knockdown cells; Figure S5: Original Western blot images corresponding to Figure 3E; Figure S6: Original Western blot images corresponding to Figure 4B; Figure S7: Original Western blot images corresponding to Figure 5A; Figure S8: Original Western blot images corresponding to Figure S3A; Figure S9: Original Western blot images corresponding to Figure 8A; Figure S10: Original Western blot images corresponding to Figure 8B; Table S1: Primers used for quantitative real-time PCR analysis; Table S2: Information on antibodies used in Western blot analysis.

## References

[1] Dai H., Ma N., Chang G., Aabdin Z.U., Shen X. (2022). Long-term high-concentrate diet feeding induces apoptosis of rumen epithelial cells and inflammation of rumen epithelium in dairy cows. Anim. Biotechnol..

[2] Aschenbach J.R., Zebeli Q., Patra A.K., Greco G., Amasheh S., Penner G.B. (2019). Symposium Review: The importance of the ruminal epithelial barrier for a healthy and productive cow. J. Dairy Sci..

[3] Meng M., Wang L., Wang Y., Ma N., Xie W., Chang G., Shen X. (2022). A High-concentrate diet provokes inflammation, endoplasmic reticulum stress, and apoptosis in mammary tissue of dairy cows through the upregulation of STIM1/ORAI1. J. Dairy Sci..

[4] Zhang B., Xie J., Li X. (2026). High-grain diet-induced ruminal acidosis triggers systemic inflammation and serum metabolic reprogramming in dairy cows. Front. Vet. Sci..

[5] Zhang K., Meng M., Gao L., Tu Y., Bai Y. (2019). Rumen-Derived Lipopolysaccharide Induced Ruminal Epithelium Barrier Damage in Goats Fed a High-Concentrate Diet. Microb. Pathog..

[6] Nishihara K., Suzuki Y., Haga S., Roh S. (2024). TLR5 ligand induces the gene expression of antimicrobial peptides and CXCL8 through IL-1β gene expression in cultured rumen epithelial cells. Anim. Sci. J..

[7] Shi J., Zhao Y., Wang Y., Gao W., Ding J., Li P., Hu L., Shao F. (2014). Inflammatory caspases are innate immune receptors for intracellular LPS. Nature.

[8] Ji X., Tong H., Settlage R., Yao W., Jiang H. (2021). Establishment of a bovine rumen epithelial cell line. J. Anim. Sci..

[9] Sarmikasoglou E., Chu L., Yue F., Faciola A.P. (2024). Effects of ruminal lipopolysaccharide exposure on primary bovine ruminal epithelial cells. J. Dairy Sci..

[10] Liu J., Xu T., Liu Y., Zhu W., Mao S.A. (2013). High-grain diet causes massive disruption of ruminal epithelial tight junctions in goats. Am. J. Physiol. Regul. Integr. Comp. Physiol..

[11] Zhan J., Gu Z., Wang H., Liu Y., Wu Y., Huo J. (2024). Rutin alleviated lipopolysaccharide-induced damage in goat rumen epithelial cells. Anim. Biosci..

[12] Wang K., Lei Q., Ma H., Jiang M., Yang T., Ma Q., Datsomor O., Zhan K., Zhao G. (2022). Phloretin protects bovine rumen epithelial cells from LPS-induced injury. Toxins.

[13] Ma Y., Elmhadi M., Wang C., Li Z., Zhang H., He B., Zhao X., Zhang Z., Wang H. (2022). Thiamine supplementation alleviates lipopolysaccharide-triggered adaptive inflammatory response and modulates energy state via suppression of NFκB/P38 MAPK/AMPK signaling in rumen epithelial cells of goats. Antioxidants.

[14] Stefanska B., Człapa W., Pruszynska-Oszmałek E., Szczepankiewicz D., Fievez V., Komisarek J., Stajek K., Nowak W. (2018). Subacute ruminal acidosis affects fermentation and endotoxin concentration in the rumen and relative expression of the CD14/TLR4/MD2 genes involved in lipopolysaccharide systemic immune response in dairy cows. J. Dairy Sci..

[15] Akira S., Takeda K. (2004). Toll-like receptor signalling. Nat. Rev. Immunol..

[16] Sakai J., Cammarota E., Wright J.A., Cicuta P., Gottschalk R.A., Li N., Fraser I.D.C., Bryant C.E. (2017). Lipopolysaccharide-induced NF-κB nuclear translocation is primarily dependent on MyD88, but TNFα expression requires TRIF and MyD88. Sci. Rep..

[17] Zhao W., Shen T., Zhao B., Li M., Deng Z., Huo Y., Aernouts B., Loor J.J., Psifidi A., Xu C. (2024). Epigallocatechin-3-gallate protects bovine ruminal epithelial cells against lipopolysaccharide-induced inflammatory damage by activating autophagy. J. Anim. Sci. Biotechnol..

[18] Liu T., Zhang L., Joo D., Sun S.-C. (2017). NF-κB signaling in inflammation. Signal Transduct. Target. Ther..

[19] Fitzgerald K.A., Kagan J.C. (2020). Toll-like receptors and the control of immunity. Cell.

[20] Jiang M., Wang K., Huang Y., Zhang X., Yang T., Zhan K., Zhao G. (2023). Quercetin alleviates lipopolysaccharide-induced cell oxidative stress and inflammatory responses via regulation of the TLR4-NF-κB signaling pathway in bovine rumen epithelial cells. Toxins.

[21] Tong Z., He W., Fan X., Guo A. (2022). Biological function of plant tannin and its application in animal health. Front. Vet. Sci..

[22] Besharati M., Maggiolino A., Palangi V., Kaya A., Jabbar M., Eseceli H., De Palo P., Lorenzo J.M. (2022). Tannin in ruminant nutrition: Review. Molecules.

[23] Patra A.K., Saxena J. (2011). Exploitation of dietary tannins to improve rumen metabolism and ruminant nutrition. J. Sci. Food Agric..

[24] Naumann H.D., Tedeschi L.O., Zeller W.E., Huntley N.F. (2017). The role of condensed tannins in ruminant animal production: Advances, limitations and future directions. Rev. Bras. Zootec..

[25] Zhang K., Qian Q., Mao Y., Xu Y., Yang Y., Chen Y., Wang X. (2021). Characterization of growth phenotypes and gastrointestinal tract microbiota in sheep fed with Caragana. J. Appl. Microbiol..

[26] Zebeli Q., Metzler-Zebeli B.U. (2012). Interplay between rumen digestive disorders and diet-induced inflammation in dairy cattle. Res. Vet. Sci..

[27] Foggi G., Terranova M., Daghio M., Amelchanka S.L., Conte G., Ineichen S., Agnolucci M., Viti C., Mantino A., Buccioni A. (2024). Evaluation of ruminal methane and ammonia formation and microbiota composition as affected by supplements based on mixtures of tannins and essential oils using Rusitec. J. Anim. Sci. Biotechnol..

[28] (2018). Laboratory Animal—Guideline for Ethical Review of Animal Welfare.

[29] Li H., Li D.-B., Niu X.-Y., Qu W. (2022). Extraction process optimization of condensed tannin from *Caragana korshinskii*. Anim. Husb. Feed. Sci..

[30] Li M., Zhu S., Huo Y., Cao Q., Deng Z., Li K., Li Y., Loor J.J., Wan J., Qi J. (2026). Microbiota-derived indole-3-acetic acid alleviates rumen epithelial barrier dysfunction during the peripartum period through AhR signaling. npj Biofilms Microbiomes.

[31] Zhang L., Xia Z., Fu J., Yang Y. (2025). Role of the rumen epithelium and associated changes under high-concentrate diets. Int. J. Mol. Sci..

[32] Xu J., Chen X., Ren J., Xu J., Zhang L., Yan F., Liu T., Zhang G., Huws S.A., Yao J. (2025). Multi-omics insights into microbiome-rumen epithelium interaction mechanisms underlying subacute rumen acidosis tolerance in dairy goats. Genome Biol..

[33] Ma X., Li M., Lu G., Wang R., Wei Y., Guo Y., Yu Y., Jiang C. (2021). Anti-inflammation of epicatechin mediated by TMEM35A and TMPO in bovine mammary epithelial cell line cells and mouse mammary gland. J. Dairy Sci..

[34] Li W., Liu H., Zhou J.-S., Cao J.-F., Zhou X.-B., Choi A.M.K., Chen Z.-H., Shen H.-H. (2012). Caveolin-1 inhibits expression of antioxidant enzymes through direct interaction with nuclear erythroid 2 p45-related factor-2 (Nrf2). J. Biol. Chem..

[35] Ekambaram S.P., Aruldhas J., Srinivasan A., Erusappan T. (2022). Modulation of NF-κB and MAPK signalling pathways by hydrolysable tannin fraction from terminalia chebula fruits contributes to its anti-inflammatory action in RAW 264.7 Cells. J. Pharm. Pharmacol..

[36] Wu S., Liao X., Zhu Z., Huang R., Chen M., Huang A., Zhang J., Wu Q., Wang J., Ding Y. (2022). Antioxidant and anti-inflammation effects of dietary phytochemicals: The Nrf2/NF-κB signalling pathway and upstream factors of Nrf2. Phytochemistry.

[37] Ma X., Wang R., Yu S., Lu G., Yu Y., Jiang C. (2020). Anti-inflammatory activity of oligomeric proanthocyanidins via inhibition of NF-κB and MAPK in LPS-stimulated MAC-T cells. J. Microbiol. Biotechnol..

[38] Krajka-Kuźniak V., Baer-Dubowska W. (2021). Modulation of Nrf2 and NF-κB signaling pathways by naturally occurring compounds in relation to cancer prevention and therapy. Are combinations better than single compounds?. Int. J. Mol. Sci..

[39] Musial C., Kuban-Jankowska A., Gorska-Ponikowska M. (2020). Beneficial properties of green tea catechins. Int. J. Mol. Sci..

[40] Crowe-White K.M., Evans L.W., Kuhnle G.G.C., Milenkovic D., Stote K., Wallace T.C. (2022). Flavan-3-ols and cardiometabolic health: First ever dietary bioactive guideline. Adv. Nutr..

[41] Rice-Evans C.A., Miller N.J., Paganga G. (1996). Structure–antioxidant activity relationships of flavonoids and phenolic acids. Free Radic. Biol. Med..

[42] Liu W., Cui X., Zhong Y., Ma R., Liu B., Xia Y. (2023). Phenolic metabolites as therapeutics in inflammation and neoplasms: Molecular pathways explaining their efficacy. Pharmacol. Res..

[43] Yang T., Datsomor O., Jiang M., Ma X., Zhao G., Zhan K. (2022). Protective Roles of Sodium Butyrate in Lipopolysaccharide-Induced Bovine Ruminal Epithelial Cells by Activating G Protein-Coupled Receptors 41. Front. Nutr..

[44] Al Mamun A., Shao C., Geng P., Wang S., Xiao J. (2024). Polyphenols targeting NF-κB pathway in neurological disorders: What we know so far?. Int. J. Biol. Sci..

