# Supplementary material for: Caragana korshinskii Tannin Attenuates LPS-Induced Sheep Rumen Epithelial Injury via TLR4/MyD88/NF-κB Signaling
Source: Biology (Basel). 2026 Jul 31;15(15):1258. doi: 10.3390/biology15151258 (PMC13465128; doi:10.3390/biology15151258)
Supplement: Supplementary file 1 [file biology-15-01258-s001.zip › biology-4424554-supplementary.pdf]

Supplementary Materials

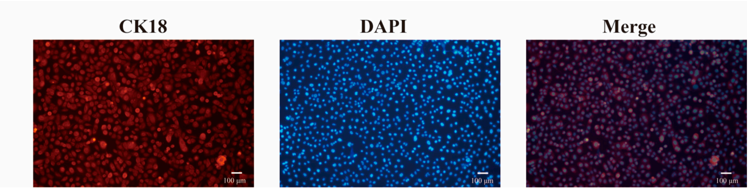

Figure S1. CK18 immunofluorescence staining supporting the epithelial identity of isolated primary sheep rumen epithelial cells.

Representative immunofluorescence images of primary sheep rumen epithelial cells stained for cytokeratin 18 (CK18). Nuclei were counterstained with DAPI. The merged image supported the epithelial identity of the cultured cells. Scale bars = 100 μm.

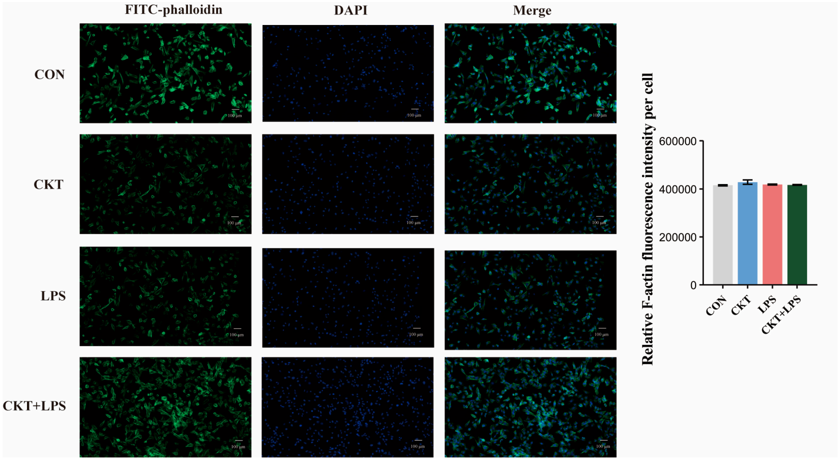

Figure S2. Effects of CKT pretreatment on F-actin organization in LPS-challenged primary sheep rumen epithelial cells.

Representative fluorescence images of F-actin organization in primary sheep rumen epithelial cells. F-actin was stained with FITC-phalloidin, and nuclei were counterstained with DAPI. Scale bars = 100 μm.

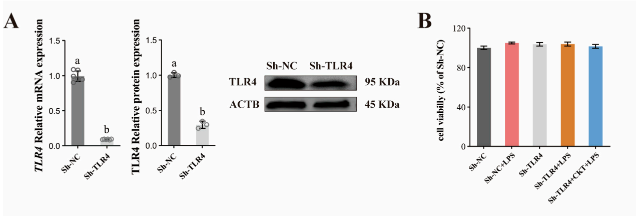

Figure S3. Verification of TLR4 knockdown efficiency and effects on cell viability in a sheep rumen epithelial cell line.

(A) Relative TLR4 mRNA expression, relative TLR4 protein expression, and representative Western blot bands showing TLR4 knockdown efficiency in cells transduced with TLR4-targeting shRNA (Sh-TLR4) or negative-control shRNA (Sh-NC). Data are presented as means ± SEM. For qRT-PCR analysis, n = 6; for Western blot analysis, n = 3. Different lowercase letters indicate significant differences between groups, $P < 0.05$. (B) Cell viability of Sh-NC, Sh-NC+LPS, Sh-TLR4, Sh-TLR4+LPS, and Sh-TLR4+CKT+LPS cells.

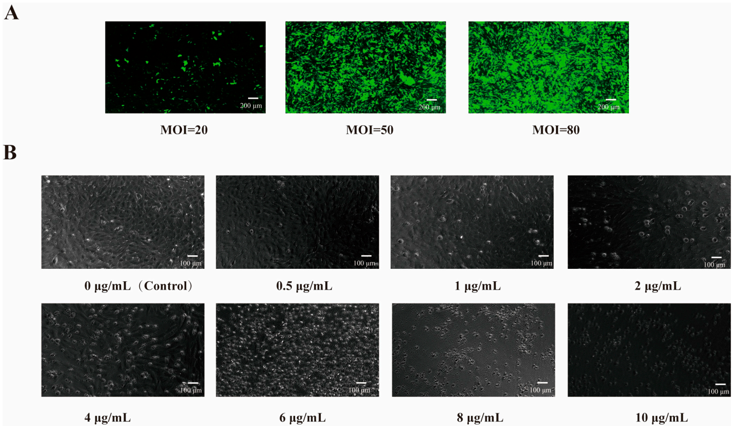

Figure S4. Optimization of lentiviral transduction conditions and puromycin selection for stable TLR4-knockdown cells.

(A) Representative fluorescence images of a sheep rumen epithelial cell line transduced with lentiviral particles at different multiplicities of infection (MOI = 20, 50, and 80). Scale bars = 200 μm. (B) Representative phase-contrast images of the sheep rumen epithelial cell line treated with different concentrations of puromycin, including 0, 0.5, 1, 2, 4, 6, 8, and 10 μg/mL. Scale bars = 100 μm.

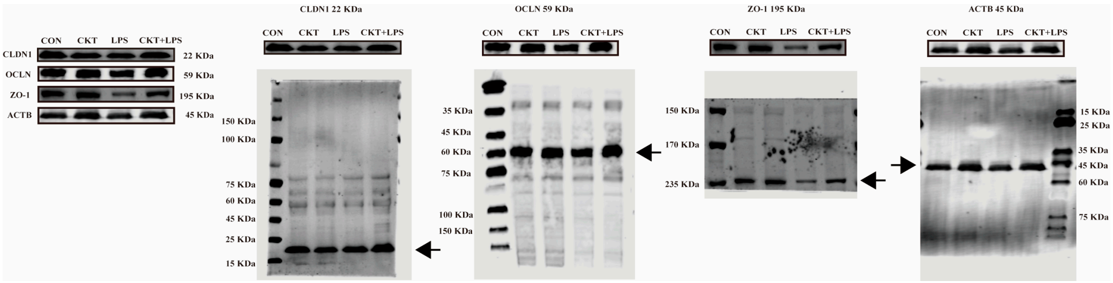

Figure S5. Original Western blot images corresponding to Figure 3E.

Complete available membrane-section images showing CLDN1, OCLN, ZO-1, and ACTB, with molecular weight markers indicated.

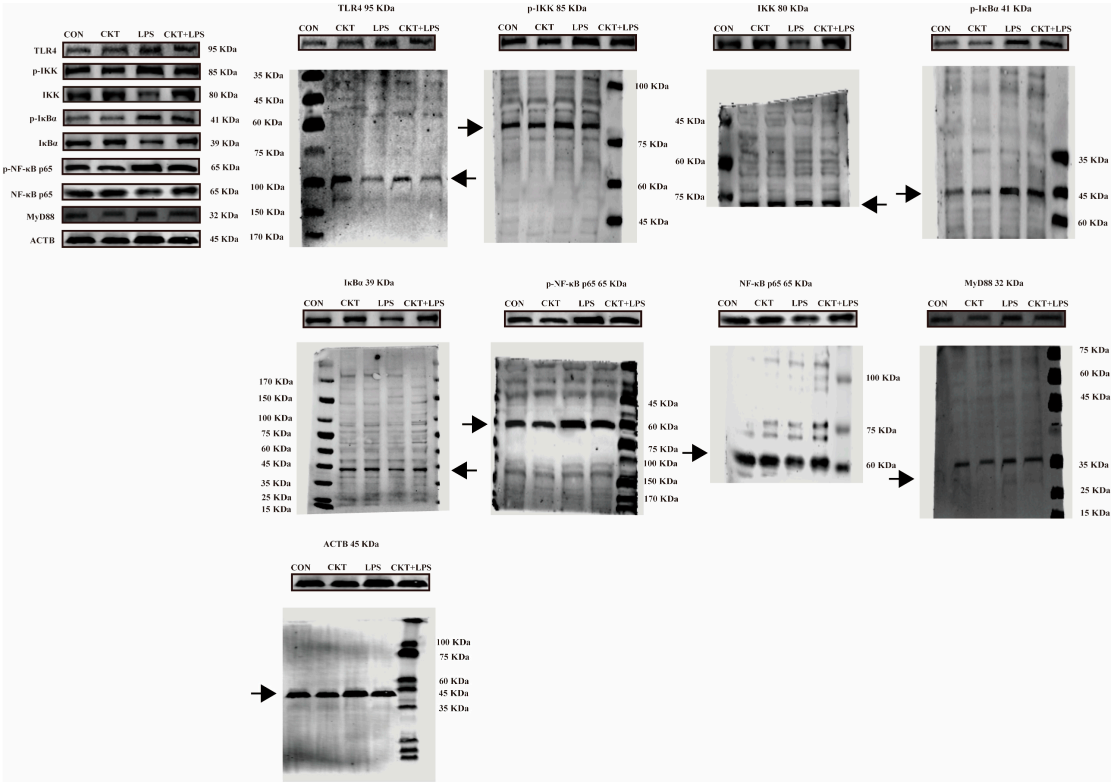

Figure S6. Original Western blot images corresponding to Figure 4B.

Complete available membrane-section images showing TLR4, MyD88, IKK, p-IKK, IκBα, p-IκBα, NF-κB p65, p-NF-κB p65, and ACTB, with molecular weight markers indicated.

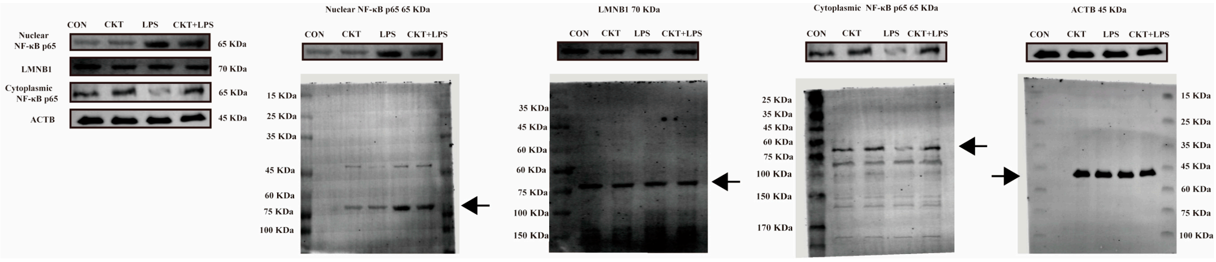

Figure S7. Original Western blot images corresponding to Figure 5A.

Complete available membrane-section images showing nuclear NF-κB p65, LMNB1, cytoplasmic NF-κB p65, and ACTB, with molecular weight markers indicated.

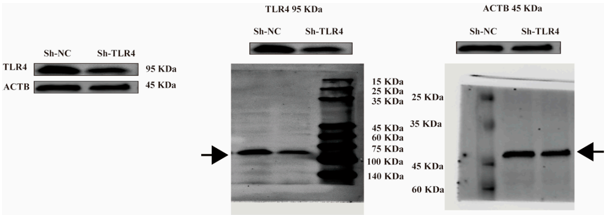

Figure S8. Original Western blot images corresponding to Figure S3A.

Complete available membrane-section images showing TLR4 and ACTB, with molecular weight markers indicated.

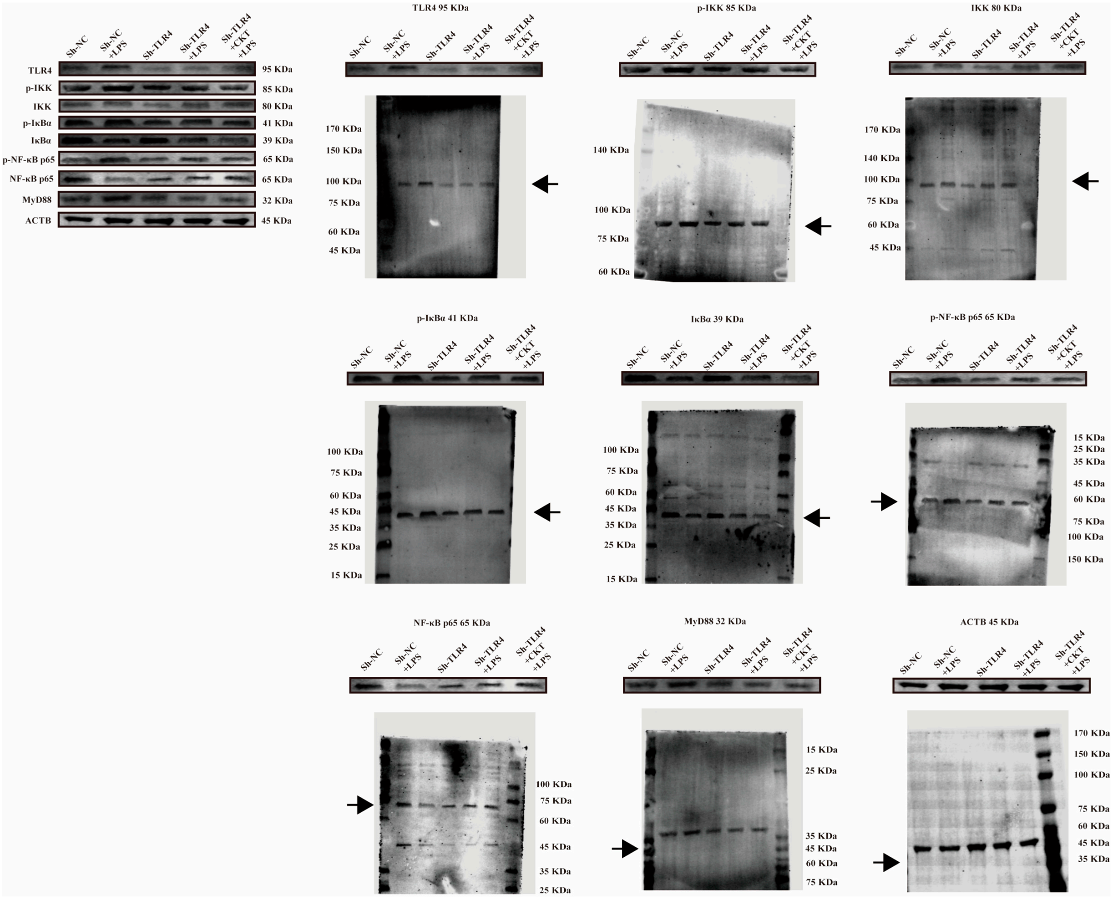

Figure S9. Original Western blot images corresponding to Figure 8A.

Complete available membrane-section images showing TLR4, MyD88, IKK, p-IKK, IκBα, p-IκBα, NF-κB p65, p-NF-κB p65, and ACTB, with molecular weight markers indicated.

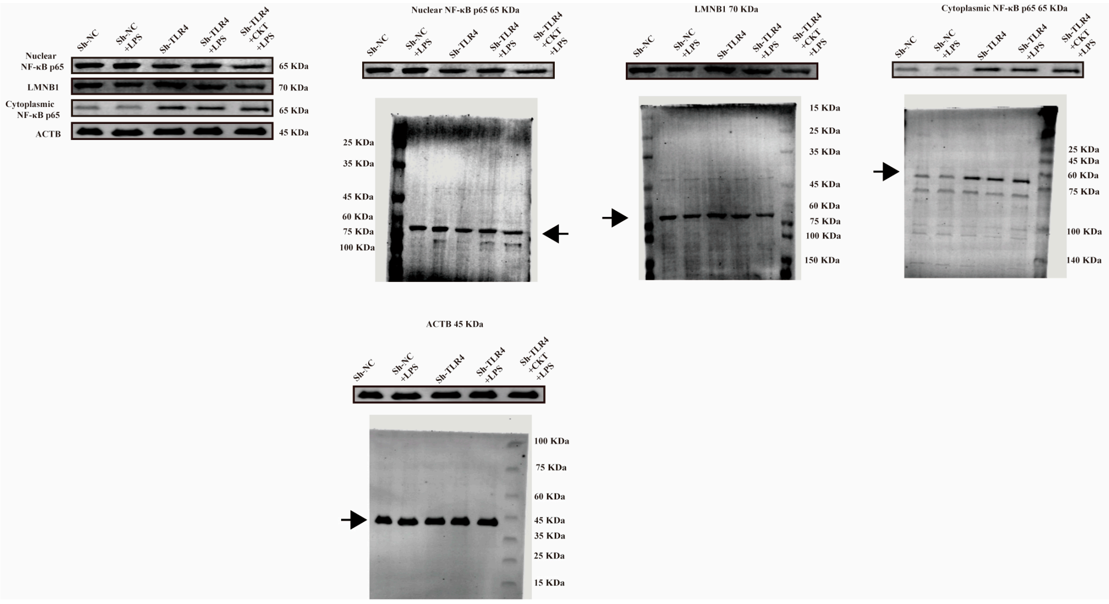

Figure S10. Original Western blot images corresponding to Figure 8B.

Complete available membrane-section images showing nuclear NF-κB p65, LMNB1, cytoplasmic NF-κB p65, and ACTB, with molecular weight markers indicated.

Table S1. Primers used for quantitative real-time PCR analysis.

Gene	Forward primer (5′→3′)	Reverse primer (5′→3′)	Product size (bp)	Accession number
ACTB	TGAGGAGCACCCTG TG	GTCTCAAACATGAT CTGGGT	84	NM_007393.5
OCLN	CTCAGGTCGTCCAGT CTTCTCAG	TCACCACCAAGTCT CCGCTTAC	182	XM_012096797.5
CLDN1	GCTGCTGCTTCTCTC TGCCTTC	AGGTGCTGGCTTGG GATAGGG	110	NM_001185016.1
CLDN3	TTCATCGGCAGCAG CATCATCAC	CAGCAGCGAGTCGT ACACCTTG	222	XM_004020967.5
CLDN4	TCATCGGCAACATC GTCAC	CAGCAGCGAGTCGT ACACCTTC	109	XM_005697785.2
CLDN7	CCTGCGGCTTCTAAT CTTATCGG	GCAGTCCTGGCTCTC GTCTC	184	NM_001185018.1
ZO-1	CGAGCAGACGCAGA AAA	GGCAGAAGATTGTG GTTGA	123	XM_015101953.2
JAM1	TGACCACAAGTAGA CCACCAGAG	ACAGCAGGAAGGA GCCAAGAAG	208	XM_004002677.5
GPx1	AACGTAGCATCGCT CTGAGG	CAAACTGGTTGCAC GGGAAG	115	XM_004018462.5
CAT	GGCTCTGTGGTGCTC TGTGTTC	TGGCGGTGAGTGTC AGGATAGG	282	XM_060400055.1
HO1	CAGGCCACCAAGTG CTATGT	CAGGGCCTTCTGAG CAATCT	141	XM_027967703.3
SOD	TTGGAGACCTGGGC AATGTG	CTGCCCAAGTCATCT GGTCT	142	NM_001145185.2
NQO1	TCTGGCCAATTCAG AGTGGCA	CAGGATCTGAACTC GGGCAT	110	XM_004015102.6
CASP1	GGTGGTATGGGTAA ACGATTCAGTAG	TGTGGGCTTTCTTAA TGGCATCAG	95	XM_004015962.6
CASP3	AGCAAGTTTCTTCAG AGGGGACTG	AGAACCACTGTCTG TCTCAATACCAC	118	XM_015104559.3
CASP8	TGATGCTATTCCAGA GGCTCCAAG	GTGTCCAAGTAGTT AAGCAGCAGATC	118	XM_012142477.5
CASP9	ACTGCTGCGTGGTG GTCATC	TTCTCTCAACGGAC ACAGGACATC	105	XM_001166184.1
Bax	CCAAGAAGCTGAGC GAGTGTCTG	GTGTCCACGGCTGC GATCATC	88	XM_027978592.3
Bcl-2	GCACCTGACGCCCT TCACC	CACACATGACCCCT CCGAACTC	119	XM_012103831.5

IL-1β	GAAGAGCTGCACCC AACACCTG	CGACACTGCCTGCC TGAAGC	106	NM_001009465.2
IL6	GCTGCTCCTGGTGAT GACTTCTG	AGTAGTCTGCTTGG GGTGGTGTC	87	NM_001009392.1
TNFα	CTGGCGGAGGAGGT GCTCTC	GGAGGAAGGAGAA GAGGCTGAGG	85	NM_001024860.1

Table S2. Information on antibodies used in Western blot analysis.

Target protein	Cat. #	Expected/observed molecular weight	Dilution	Category	Manufacturer
Occludin/OCLN	27260-1-AP	~65 kDa	1:500	Primary antibody	Proteintech
Claudin1/CLDN1	13050-1-AP	~22–23 kDa	1:500	Primary antibody	Proteintech
ZO-1	21773-1-AP	~220 kDa	1:500	Primary antibody	Proteintech
TLR4	19811-1-AP	~95 kDa	1:500	Primary antibody	Proteintech
IKK	15649-1-AP	~80 kDa	1:500	Primary antibody	Proteintech
Phospho-IKK	2697	~80 kDa	1:1000	Primary antibody	Cell Signaling Technology
IκBα	10268-1-AP	~39 kDa	1:500	Primary antibody	Proteintech
Phospho-IκBα	82349-1-RR	~39 kDa	1:500	Primary antibody	Proteintech
NF-κB p65	10745-1-AP	~65 kDa	1:500	Primary antibody	Proteintech
Phospho-NF-κB p65	82335-1-RR	~65 kDa	1:500	Primary antibody	Proteintech
MyD88	23230-1-AP	~33 kDa	1:500	Primary antibody	Proteintech
ACTB/β-actin	4970	~42 kDa	1:1000	Primary antibody	Cell Signaling Technology
Lamin B1/LMNB1	12987-1-AP	~66 kDa	1:1000	Primary antibody	Proteintech
Anti-rabbit IgG (H+L), DyLight 800 Conjugate	5151s	N/A	1:10000	Secondary antibody	Cell signaling technology
